# Ghrelin and the Control of Energy Balance in Females

**DOI:** 10.3389/fendo.2022.904754

**Published:** 2022-07-15

**Authors:** Andrea Smith, Barbara Woodside, Alfonso Abizaid

**Affiliations:** ^1^ Department of Neuroscience, Carleton Unversity, Ottawa, ON, Canada; ^2^ Stress, Trauma and Relience (STAR) Work Group Carleton University, Ottawa, ON, Canada

**Keywords:** ghrelin, GHSR, steroid hormones, estradiol, feeding, energy balance, stress, food reward

## Abstract

Ghrelin is considered one of the most potent orexigenic peptide hormones and one that promotes homeostatic and hedonic food intake. Research on ghrelin, however, has been conducted predominantly in males and particularly in male rodents. In female mammals the control of energy metabolism is complex and it involves the interaction between ovarian hormones like estrogen and progesterone, and metabolic hormones. In females, the role that ghrelin plays in promoting feeding and how this is impacted by ovarian hormones is not well understood. Basal ghrelin levels are higher in females than in males, and ghrelin sensitivity changes across the estrus cycle. Yet, responses to ghrelin are lower in female and seem dependent on circulating levels of ovarian hormones. In this review we discuss the role that ghrelin plays in regulating homeostatic and hedonic food intake in females, and how the effects of ghrelin interact with those of ovarian hormones to regulate feeding and energy balance.

## Introduction

Obesity is a major risk factor for the development of metabolic disorders, such as diabetes mellitus and cardiovascular disease (1). Obesity is also co-morbid with mental health disorders like depression, anxiety and some eating disorders that have a higher incidence in biologically female individuals (2, 3). Importantly, metabolic disorders are thought to emerge from changes in the functioning of endocrine and neural mechanisms associated with the regulation of energy homeostasis (4). In women, these regulatory processes are influenced by the cyclic release of estrogen and progesterone during the reproductive cycle (5). Furthermore, the fluctuations and decline of ovarian hormones that occur during menopause are associated with an increased risk of obesity and other metabolic disorders (6, 7). While the relationship between ovarian steroid hormones and satiety metabolic peptides like leptin has been examined previously (5, 8, 9), most studies on the role othe orexigenic hormone ghrelin in energy balance comes from research conducted on male rodents. Here we review studies investigating the interaction between ovarian hormones and ghrelin in the regulation of feeding and energy balance in female mammals.

## Ghrelin: A Potent Stimulator of Food Intake

Ghrelin, a 28-amino acid peptide secreted from the stomach, binds to the growth hormone secretagogue receptor (GHSR) (10, 11). Given previous work demonstrating the orexigenic and adpogenic effects of growth hormone secretagogues (12), it was not surprising to find that ghrelin was a potent stimulator of caloric intake and promoted carbohydrate metabolism and fat accumulation (11, 13, 14). Ghrelin is secreted from the stomach during states of negative energy balance and in anticipation of regularly scheduled meals. As food is consumed, ghrelin secretion declines to baseline levels (15–17). Two forms of ghrelin are present in circulation, des-acyl and acyl-ghrelin. Des-acyl ghrelin was often referred to as the inactive form of the peptide, because it does not activate the GHSR (18). There is work, however, that suggests this peptide has other biological actions (19). Acyl-ghrelin, hereafter referred to as ghrelin unless specified, is produced following octanolyation of desacyl-ghrelin *via* the enzyme ghrelin o-acyltransferase (GOAT) (20). Once acylated, ghrelin can effectively induce its biological effects *via* activation of the GHSR.

**Figure 1:**
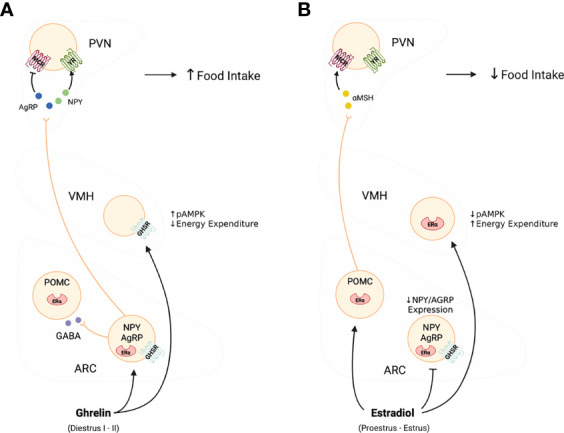
Effects of ghelin **(A)**, and estradiol **(B)** on the activity of the melanocortin system. As shown in Panel **(A)** in this diagram, ghrelin selectively stimulates the activity and release of NPY/AGRP neurons to promote food intake and adiposity. In contrast, as shown in Panel **(B)**, estradiol targets both NPY/AGRP neurons and POMC to ultimately decrease food intake and increase metabolic rate. The effects of estradiol are linked direct inhibition of NPY/AGRP neurons while stimulating POMC neurons and favouring the release of α-MSH, and together they oppose the effects of ghrelin.

Ghrelin promotes food intake and metabolic changes in part through GHSR activation within the arcuate nucleus (ARC) of the hypothalamus (13, 14) (See **Figure 1**). Within the ARC, Neuropeptide Y (NPY) and agouti-related peptide (AgRP) neurons promote increases in food intake and decreases in energy expenditure, whereas neurons producing pro-opiomelanocortin (POMC) and cocaine and amphetamine-regulated transcript (CART) promote satiety and increase metabolic rate (4, 21). The NPY/AgRP neurons also release γ-aminobutyric acid (GABA) to inhibit POMC/CART cells as well as cells in the brain stem parabrachial nucleus to produce an overall orexigenic effect (22). Mice with AgRP-specific deletion of the GABA transporter display reduced body weight and food intake suggesting that GABA release from AgRP and NPY neurons in the ARC plays a major role in inhibiting the POMC/CART satiety-inducing pathway (23, 24). Upon release, NPY directly stimulates feeding and decreases energy expenditure *via* activation of NPY 1 and 5 receptor subtypes expressed throughout a number of hypothalamic and extrahypothalamic regions (4). Secretion of AgRP stimulates feeding by acting as a competitive antagonist of α-melanocyte-stimulating hormone (α-MSH), at melanocortin receptors 3 and 4 (MCR3/4) (21). The routes through which ghrelin accesses the ARC and other brain regions have been the subject of much debate [reviewed in (25)]. However, the ARC is strategically situated bilaterally at the bottom of the third ventricle and above the median eminence, a region with direct access to circulating peripheral metabolic signals, including ghrelin and leptin (4). Evidence suggests that the fenestrated capillaries of the median eminence allow for passive movement of ghrelin into the ARC and perhaps to other hypothalamic and extrahypothalamic regions (26–28).

In addition to the ARC, peripheral ghrelin binds to GHSR expressing cells in brain stem regions that regulate feeding in response to rapid changes in metabolic signals like glucose and fatty acids in blood, and that integrate hormonal and neural signals coming from the gut *via* the ascending branch of the vagus nerve (29). Ghrelin receptors are located in the nodose ganglia which transmit sensory information from the alimentary tract (30), and their target the nucleus of the solitary tract (NTS) (29). In addition to GHSR, the NTS expresses receptors for most metabolic signals including estrogen, leptin, and cholecystokinin (CCK). Noradrenergic cells in the NTS integrate ascending vagal stimulation and project to other brain stem regions such as the parabrachial nucleus (PB), which when stimulated, are important for a reduction in appetite through hypothalamic corticolimbic projections including projections to the ARC (31–34). Indeed, peripheral ghrelin decreases afferent vagal discharge in the NTS whereas CCK increases discharge, suggesting that vagal stimulation of NTS targets represents an integration of orexigenic and anorexigenic signals (35, 36). Increases in peripheral ghrelin leads to activation of NTS noradrenergic neurons that project to the ARC and disrupting this pathway prevents peripheral ghrelin-induced feeding (34).

**Figure 2:**
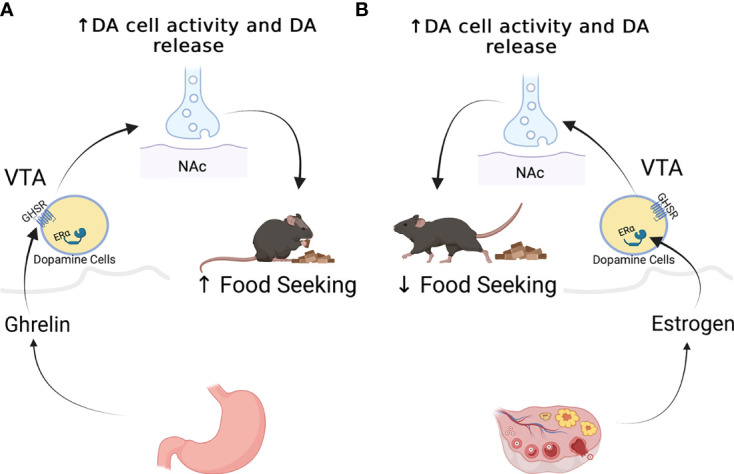
Effects of ghrelin **(A)**, and estradiol **(B)** on the mesolimbic dopaminergic system. Ghrelin stimulates the activity of dopamine neurons and the release of dopamine at target regions to promote food seeking behaviors in male mice **(A)**. While studies ghave not done similar work on females, estradiol appears to increase the activity of dopamine cells, but appears to decrease food seeking behaviors **(B)**.

Ghrelin targets other brain regions to modulate food preference and food motivation. These effects are believed to be mediated *via* GHSR expression throughout brain regions important in reward regulation [For review see (37)]. This system comprises dopaminergic neurons originating in the ventral tegmental area (VTA) that project to the nucleus accumbens, amygdala, hippocampus, and prefrontal cortex (38) . VTA dopaminergic neurons express the GHSR (29, 39, 40), and intra-VTA ghrelin administration in rodents results in increased DA release into NAcc as well as increasing food intake and food motivation as measured by increased lever presses for food, or in response to contextual or specific cues such as the presence of a light associated with food availability (39, 41–44) (see **Figure 2**). In human functional MRI studies, intravenous ghrelin administration coupled with the presentation of images of palatable food increased neural activity within the VTA along with self reports of cravings (45).

## Ghrelin and Energy Balance in Females: Interaction With Steroid Hormones

Despite the fact that there are clear sex differences in patterns of food intake, and energy expenditure and emerging evidence of sex differences in the brain regions subserving feeding (see (46)for review) most of the studies of ghrelin’s effects on metabolism have been carried out in male subjects. Of the studies carried out in females, most have focused on the effects of ghrelin on the somatotrophic and reproductive axes along with the effects of reproductive hormones on ghrelin synthesis (See **Table 1**). For example, GHSR are localised in the pituitary where ghrelin stimulates growth hormone secretion (10, 11). Interestingly, adult (36 week old) female transgenic mice with a targerted deletion of the ghrelin gene (*Ghrl^-/-^* mice) show decreased pulsatile grothw hormone, whereas *Ghrl^-/-^* male mice do not show these growth hormone disturbances (54). The GHSR is also expressed in multiple sites along the hypothalamo-pituitary ovarian axis including the medial preoptic area (MPOA), kisspeptin neurons in the ARC, gonadotrophs in the pituitary, and the ovaries (29, 52, 53). Ghrelin acting on GHSR in the MPOA of estradiol-primed rats decreases luteinizing hormone (LH) release (55–57) and can also decrease progesterone secretion directly by acting on GHSR within corpus luteum cells (58, 59). These effects have led to the suggestion that ghrelin, acting as a signal of food shortage, suppresses the reproductive axis (60).

**Table 1:** List of publications looking at the effects of ghrelin on homeostatic feeding, and the interaction of these effects with estrogen.

	Effect of…	Results Summary	References
**Homeostatic Feeding**	**Estrus Cycle**	Reduced food intake and meal size during proestrus and estrus	(47) (review)
		Reduced ghrelin sensitivity, no orexigenic response during proestrus and estrus	(48)
	**Ghrelin or GHSR deletion**	Reduced food intake, body weight, and adiposity	(49) (50)
	**Ovariectomy**	Increased ghrelin and ghrelin sensitivity	(48)
	**Ovariectomy + Estradiol Replacement**	Reduced food intake and body weight	(51)
		Increased GHSR expression^1,2,^ and ERα colocalization in ARC kisspeptin neurons^1^	^1^ (52) ^2^ (53)
		Unknown: Effects on circulating ghrelin	
	**Ovariectomy + Progesterone Replacement**	Unknown: Effects on circulating ghrelin or GHSR expression	
	**GHSR deletion + Ovariectomy**	No increase in food intake or body weight	(48)
		Unknown: Effects on circulating ghrelin	

Conversely, there is also evidence that ovarian hormones modulate ghrelin secretion. Estrogen receptor alpha (ERα) is expressed in ghrelin producing cells in the stomach of rats, and locally produced estrogen acts directly on these ghrelin producing cells to stimulate ghrelin expression and secretion (61, 62). Notably, ghrelin expression or secretion were altered three weeks after gonadectomy in either male or female rats suggesting that this effect is independent of cirulating levels of estrogen (62), although others have reported a transient increase in circulating ghrelin, after ovariectomy (48, 61). Nevetheless, it is unclear if these studies measured total, des-acyl or ghrelin, and this distinction is important for assessing the functional consequences of such changes, because an increase in total ghrelin does not necessarily entail an increase in ghrelin and hence greater orexigenic drive. Böchers et al. recently confirmed that female rats have higher circulating ghrelin levels than males, and that ovariectomy resulted in reduced acyl-ghrelin concentrations using a well characterized acyl-ghrelin assay. These authors also and also showed that females have lower plasma concentrations of the liver anitimicrobial peptide-2 (LEAP-2), an endogenous antagonist to the GHSR (63, 64).

In women, chronic oral estrogen treatment increases plasma ghrelin concentrations but this effect is not seen after acute or short term transdermal administration of estrogen (65, 66). A number of studies have reported higher circulating levels of ghrelin in females than in males (67, 68). As with rodent work, most of these studies either measured total ghrelin (67) or did not define whether total ghrelin or ghrelin were measured (68). For example Kaur et al, found that in mice total ghrelin was higher in late pregnant mice than in nonpregnant controls but that ghrelin levels were unchanged whereas in mid pregnancy ghrelin levels were lower while total ghrelin levels were unchanged (69). These data suggest that changes in GOAT activity and/or rate of metabolism of acyl ghrelin make important contributions to sex differences in circulating levels of ghrelin.

Ghrelin secretion can be enhanced directly by estradiol treatment, but the interaction between these two hormones is complex. Indeed, while estrogen increases the secretion of ghrelin, it also has metabolic effects that are opposite to those of ghrelin (46). Food intake varies across the reproductive cycle in many mammals and is lowest in the periovulatory phase when estradiol levels peak. Studies of the effects of estradiol replacement in ovariectomized rats suggests that the tonic moderate levels of estradiol seen in the follicular phase of the cycle are sufficient to decrease food intake and these are further reduced by the peak levels of E seen in the periovulatory phase (46). The estradiol-induced reduction in food intake is related to a decrease in meal size, but not frequency, suggesting that part of the anorectic effect of estradiol are associated with greater sensitivity to satiety signals during the periovulatory period (46, 70). Consistent with this, estradiol facilitates the satiating effects of oxytocin, leptin, CCK, and GLP-1 (32, 47, 71). The effects of estrogen on energy balance are not limited to food intake, as estrogen also stimulates energy expenditure (72). This is reflected in increased oxygen consumption, that is particularly evident during the dark phase of the light/dark cycle of ovariectomized females receiving estradiol treatment (73). In addition, Giles et al. reported that the respiratory exchange ratio (RER) declines during proestrus and estrus, supporting a role for estrogen in fuel utilization (74) and consistent with the hypothesis that estradiol promotes the utilization of fats as a source of energy. In sum, this evidence suggests that the effects of estrogen on metabolism are opposite to those of ghrelin so that high circulating levels of estrogen are associated with decreased food intake, increased energy expenditure and utilization of fat rather than carbohydrate.

In the brain, estradiol influences food intake and energy expenditure in regions that overlap with those targeted by ghrelin and these include the NTS, ARC and other hypothalamic nuclei with rich expression of estrogen receptors (ERs) and GHSR (75, 76). Stimulation of estrogen receptor α (ERα) in the NTS potentiates CCK induced Fos-ir (46). ICV treatment with estradiol, or with a selective ERα agonist increases the activity of POMC neurons in the ARC and decreases food intake in intact male and female mice (8), and deletion of ERα from POMC neurons decreases food intake but has no effect on either energy expenditure (77). There is also evidence that NPY/AgRP neurons of the ARC play a critical role in estrous cycle variation in food intake. Estradiol reduces the activity of NPY/AgRP neurons under both free feeding and fasted conditions (78, 79) and NPY/AgRP mRNA expression in the ARC of female mice varies with the phase of the cycle being lowest in the proestrus/estrus phase (78).

In addition to a direct effect on POMC and NPY/AgRP neurons, estradiol also influences energy expenditure by modulating the responses to NPY and AgRP, and α-MSH at their projection sites such as the ventromedial hypothalamus (VMH), paraventricular hypothalamus (PVN), dorsomedial hypothalamus (DMH) and lateral hypothalamus (LHA), all regions that express ERs and GHSR (29, 75, 76). Of these, the VMH is a particularly critical region for convergence of estrogen and ghrelin signalling. Ghrelin can directly stimulate food intake when delivered into the VMH, and may do this through activation of nutrient sensing mechanisms that include activation of AMPK and mTOR (80). In contrast, estradiol inhibits AMPK activity in the VMH (81). Silencing ERα in the VMH of mice and rats resulted in a transient increase in food intake, but a dramatic decrease in energy expenditure (82), an effect that was recapitulated when ERα was deleted specifically from SF-1 expressing neurons in the VMH (77). This effect of ERα activation is likely mediated by modulation of activity of a pathway projecting from the VMH to serotonergic neurons in the dorsal raphe nucleus a pathway important for the regulation of brown fat thermogenesis (BAT) and locomotor activity (83). While no data exist to support ghrelin’s action on this specific pathway to modulate BAT, yet central ghrelin delivery does reduce sympathetic activity reqired to stimulate BAT and reduces the activity of uncoupling protein 1 (UCP1), a marker of BAT (84–86). It is therefore likely that, as in the ARC, ghrelin and estrogen have opposite effects on VMH cells and that there are sex differences in these effects, but this requires further examination.

Overall, the data above would support the notion that estrogen directly increases the secretion of ghrelin, but strongly opposes this hormone’s orexigenic and metabolic effects. Indirect evidence for this was first observed in some of the first studes conducted on transgenic mice lacking ghrelin or GHSR. A study using *Ghrl^-/-^* mice showed that male *Ghrl^-/-^* mice gained less weight when placed on a high fat diet compared to wild type (WT) mice (Wortley et al., 2005). While females were also studied, the authors simply stated that females, regardless of the genotype, were resistant to weight gain when fed the same high fat diet. In a parallel study using mice with targeted deletion of the GHSR (*Ghsr^-/-^* mice), male and female *Ghsr^-/-^* mice and their WT littermates were followed from week 4 to determine their metabolic phenotype. When fed a normal diet, *Ghsr^-/-^* female mice gained less weight and accumulated less fat than their WT littermates, whereas no differences were observed in males. When fed a high fat diet, male and female *Ghsr^-/-^* mice gained less weight and accumulated less fat than WT control mice, but these effects appeared to be more robust in female mice (49). These data suggested a greater contribution of ghrelin to food intake in female than in male mice.

Nevertheless, Clegg et al. reported soon after that female rats required higher doses of peripheral ghrelin than males to stimulate food intake (48). This sex difference was eliminated after ovariectomy, with females showing feeding responses to similar doses as those that were effective in males. When exogenos estradiol was given to ovariectomized rats, these again required higher doses of ghrelin to stimulate a significant increase in feeding. Clegg et al. also demonstrated that the orexigenic effects of both peripheral and icv ghrelin administration fluctuated across the estrous cycle in rats with ghrelin treatment being more effective in stimulating feeding during diestrus, when plasma estradiol concentrations are low, but ineffective with the same dose during proestrus and estrus when estradiol concentrations are higher (48, 87). Finally, this study showed that the typical increase in food intake and body weight that accompanies ovariectomy in many species was not seen in ovariectomized GHSR-null mice (48). Overall this paper suggests that ghrelin does make an important contribution to energy balance in female rats but only in the absence of estrogen.

One way in which estradiol could alter the effectiveness of ghrelin would be to regulate the expression of GHSR in hypothalamic and extrahypothalamic brain regions, and hence decrease ghrelin sensitivity. Few studies, however, have examined this possibility. Moreover, studies thoroughly examining sex differences in central GHSR expression have not been conducted. Early studies showing GHSR mRNA expression clearly point to NPY neurons as being the main subpopulation of ARC neurons containing GHSR, but some studies report the proportion of NPY cells expressing GHSR mRNA in female rats being as high as 94%, whereas others show GHSR expression in NPY neurons as low as 16% in males (88, 89). Using quantitative polymerase chain reaction (qPCR), a recent study compared GHSR mRNA expression in the ARC and amygdala of male and female ad lib fed mice and mice that were fasted overnight. Female mice showed higher overall GHSR expression in both of these regions, especially in the amygdala (63). In another study, the issue of ghrelin sensitivity was examined indirectly by measuring Fos immunoreactivity (Fos-ir) in response to icv ghrelin infusions in female rats at different phases of the cycle. More Fos-ir was induced in the ARC following icv administration of ghrelin in female rats in the diestrous phase than in the proestrus phase of the cycle but, no changes in GHSR1a expression in the ARC as a function of phase of the cycle were observed in this study (90). In contrast to NPY neurons, GHSR is only expressed in few POMC neurons within the ARC, suggesting that ghrelin primarily enhances orexgenic drive. Importantly, POMC neurons express receptors for, and respond to estradiol, suggesting that estradiol may oppose ghrelin levels directly by inhibiting NPY neurons and indirectly by stimulating POMC neurons (8, 91)

Increases in GHSR expression have also been observed in cells of the ARC that colocalize with ERα when ovariectomized rodents are treated with estradiol (52, 53, 92). These cells were later identified as kisspeptin neurons, a subgroup of cells that have been linked to the control of luteinizing hormone pulsatility, but also recently implicated in the regulation of energy balance (53). Clearly there is a need for further investigation into the effect of changes in circulating estrogen on GHSR expression in the brain to ascertain in which cell groups effects occur and whether the alterations in estrogen levels seen across the cycle are sufficient to induce these changes.

## Ghrelin & Ovarian Hormones in the Regulation of Hedonic Feeding

Like ghrelin, estrogen influences the rewarding properties of food. Clinical data show that food cravings for high-fat, palatable foods, as well as binge-eating episodes,increase in the luteal phase of the menstrual cycle when estrogen levels are low and similar results have been seen in rodents (see (93)for review). Moreover, eating disorders like binge-eating, anorexia and bulimia nervosa are primarily diagnosed in females, and have been associated with dysregulation of reward circuitry (94). Importantly, sex differences have been reported in the soma size, number and proportion of dopamine cells in the VTA with females having more and larger dopamine cells in the VTA than do male rodents (see (95) for review).

**Figure 3:**
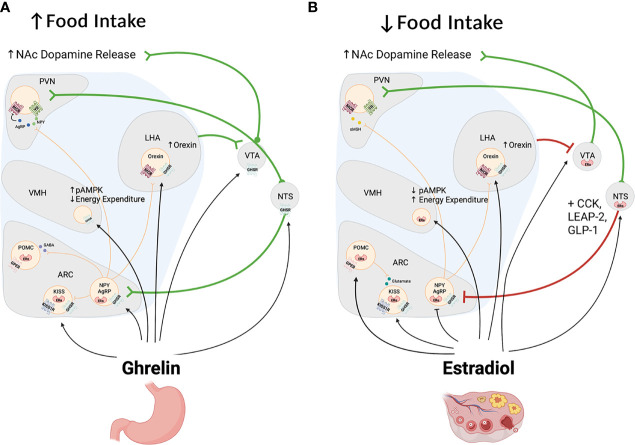
Panels **(A, B)** depict some of the brain regions affected by ghrelin **(A)** and estrogen **(B)** to regulate food intake and energy balance. As shown in Panel **(A)**, ghrelin targets hypothalamic and extrahypothalamic regions to increase homeostatic and hedonic feeding. In the hypothalamus, ghrelin acts on NPY/AGRP neurons to increase feeding and decrease energy expenditure indirectly *via* stimulation of NPY and melanocortin 3&4 receptors (MC3 and MC4 receptors) in the PVN, LHA and other regions not depicted in this figure. In addition ghrelin stimulates cells located in the NTS and VTA to increase food intake through the stimulation of ascendind catecholaminergic cells that also influence the activity of the hypothalamus and limbic system. Of these, ghrelin stimulates dopamine cells in the VTA to increase food seeking behaviors and food reward. In contrast and as shown in panel **(B)**, estradiol acts in the hypothalamus to produce effects that are opposite to those of ghrelin with an overall anorectic effects and an increase in energy expenditure. In the brain stem estrogen also decreases food intake directly and it enhances the anorectic effects of leptin and CCK. Paradoxically, estrogen, like ghrelin, stimulates the activity of dopamine cells and their release of dopamine into the nucleus accumbens [NAc; See Panel **(B)**].

In rodents, the effects of estrogen on food motivation have largely been examined using two behavioral paradigms – the food hoarding paradigm and the progressive ratio operant responding paradigm (see **Table 2**). Food hoarding refers to the behavior of collecting food without eating it and occurs in rats and hamsters (106). Hoarding behaviors in female hamsters and rats decrease during the estrous phase of the cycle increase in ovariectomized females and are reduced by estrogen or estrogen and progesterone replacement (107–109). Using the progressive-ratio operant task, a task where the number of responses required to obtain a reward (usually bar presses) increases across the testing session, Richard et al. found that free feeding female rats worked less to obtain sucrose rewards during proestrus/estrus, than in other phases of the cycle (96). Similarly, ovariectomy increases motivation to obtain a sucrose reward (110), whereas estradiol treatment in gonad-intact or ovariectomized rats reduces the motivation to obtain a chocolate flavored sucrose reward (96). Interestingly, the anorectic peptide GLP-1 was more effective in reducing the amount of bar presses to obtain food pellets when ovariectomized rats were also treated with estradiol (71). Together these data suggest that, opposite to ghrelin, estradiol decreases food seeking in female rats (see **Figure 3**).

**Table 2:** List of publications looking at the effects of ghrelin on hedonic feeding and stress, and the interaction of these effects with estrogen.

	Effect of…	Results Summary	References
**Hedonic Feeding**	**Estrus Cycle**	Reduced intake of palatable food during periods of elevated estrogen, proestrus and estrus	(96)
		Unknown: Effects of ghrelin administration on hedonic feeding throughout the estrus cycle	
	**Palatable Food Choice**	Intra-VTA ghrelin treatment increases consumption of sucrose solution	(97)
	**Operant Tests**	Intra-LH treatment with GHSR antagonist decreases sucrose rewards earned in female rats, compared to males	(98)
		Increased LH activity in orexin neurons, compared to males	(98)
		Intra-VTA estradiol treatment decreases motivation to bar press for sucrose rewards	(99)
	**Acute Stress**	Increased ghrelin, reduced food intake, compared to non-stressed females	(100)
	**Ovariectomy + Acute Stress**	Increased food intake, compared to gonad-intact, stressed females Ghrelin levels not reported	(100)
	**Chronic Stress**	Increased ghrelin^1,2^, corticosterone^1,3^, food intake^1,3^, and body weight^1,3^	^1^ (101) ^3^ (102)
		No changes to circulating ghrelin^1,2^, increased food intake^2^	^1^ (100) ^2^ (103)
	**Ovariectomy + Chronic Stress**	Reduced corticosterone and ghrelin, elevated food intake	(101)
		Increased consumption of high sucrose treat if treated with estradiol following ovariectomy	(104) (105)

As noted above food reward seeking has been linked to activity in the mesocorticolimbic dopaminergic system, and given the data discussed above, one would suspect that estradiol decreased the activity of the dopaminergic system to reduce food seeking and food reward. Nevertheless, data suggest that estradiol, like ghrelin, can act directly on dopaminergic neurons to stimulate dopamine neurotransmission. For instance, the activity of DA neurons in the VTA is higher in estrous females than it is in either males or females in diestrus and estradiol administration results in increased dopamine release in the nucleus accumbens and other targets (95, 111). There is also evidence that estrogen modulates expression of the dopamine transporter in the VTA, and/or alters its phosphorylation state to making it more susceptible to cocaine (112, 113), Consistent with these changes, the psychomotor effects of stimulants like cocaine or amphetamine are greater in the presence of estradiol (114). These effects of estrogen are thought to be mediated by intracellular and membrane bound receptors including ERα, ERβ, GPER-1 localized in VTA dopamine neurons and at their pre-and post synaptic targets (115).

Although estrogen receptors and GHSR are both expressed in multiple sites in the mesocorticolimbic system the degree of colocalization and whether there are sex differences in GHSR expression and/or colocalization with estrogen receptors is not known. In one behavioral study intra-VTA ghrelin administration in female rats increased sucrose intake and extracellular dopamine in the nucleus accumbens, confirming that ghrelin stimulates the reward system in a way similar to males (97). The dose used in this study was, however, at least double the dose needed to produce similar effects in males, but it is not clear if lower doses were used unsuccessfully in females. Also in female rats, infusions of the GHSR antagonist JMV2959 into the VTA did not affect food intake but did decrease alcohol consumption following exposure to the intermittent alcohol access paradigm, suggesting that full GHSR signalling in the VTA is required for the reinforcing effects of alcohol but not food (116).Whether further disruption of GHSR signalling is required to affect food is required and whether the effects described above vary across all stages of the estrous cycle has yet to be investigated. It is also unclear whether intra-VTA ghrelin or GHSR antagonist treatment leads to different levels of dopamine release at different phases of the estrous cycle.

Ghrelin also modulates food reward by acting at sites targeted by VTA dopamine cells and/or that send projections ot the VTA to modulate dopamine cell activity. For instance, intra nucleus accumbens (NAc) ghrelin administration increases feeding in male rats (42). Ghrelin administration into the LHA increases food intake and increased food-seeking behaviors in male and female rats. Importantly, in the current context inhibition of LH GHSR activity using the GHSR antagonist YIL-781, decreased the amount of sucrose rewards earned by female but not male rats (98). Even a 10% knock down of GHSR expression in LHA was sufficient to prevent the effects of ghrelin on reward seeking behaviors without affecting overall food intake in females (98). Similarly, estradiol acts directly on the NAc to increase dopamine release (117). In all ghrelin, and estrogen seem to prodce the same effects on the mesolimbic dopaminergic system all the while having opposite food reward feeding responses.

Recent work using a behavioral model that increases binging in mice shows that ghrelin and its receptor are implicated in the processes associated with overconsumption of a palatable hypercaloric diet that is available only during a restricted time. In this model mice have free access to a normal chow diet, and receive restricted (2-4 hours) access to a high calorie diet every other day during the light phase of the light/dark cycle. Male *Ghsr^-/-^* mice placed under this paradigm show decreased binging of the high calorie diet and reduced total caloric intake compared to WT mice (118). A similar study recently used a similar paradigm this time using *Ghrl^-/-^* female mice. Results from this study showed that female *Ghrl^-/-^* mice consumed less calories of the chow diet on binge and non-binge days, and despite binging on the high calorie diet on binge days, showed reduced locomotor activity, weight gain and lean body mass compared female WT mice (119). While it is unclear if these effects are mediated by decreased activity of reward circuitry in *Ghrl^-/-^* mice, studies using male *Ghsr^-/-^* mice in the same model suggest that this is the case (118).

## Ghrelin & Ovarian Hormones: Stress-Induced Feeding

The relationship between stress and feeding is complex, with some stressors decreasing feeding while others increasing feeding. Overall, it appears that although acute exposure to a mild stressor, such as tail pinch in rodents, generates a transient feeding response, similar exposure to stronger stressors have an anorectic effect (120, 121). Chronic exposure to unpredictable stressors also induces a decrease in food intake whereas chronic stress paradigms in which stress exposure is predictable increases food intake, and in some cases is associated with weight gain and increased adiposity (120, 121). That the ability to anticipate a stressor leads to increases in food intake suggests that stress-induced feeding is a method of coping with stress, and there is considerable evidence suggesting that ghrelin may play a role in this effect (100, 103, 122) but very little data on whether these responses are sexually differentiated.

Chronic social defeat stress (CSDS) is one example of a chronic stressor that induces increases in food intake. Male mice, exposed to social defeat daily for a period of 21days increase food intake by 10-15% compared to control mice, and this is accompanied by weight gain and adiposity (123–125). These effects are associated with an increase in circulating ghrelin (123, 125) and specifically with ghrelin actions in the ARC. *Ghsr^-/-^* mice show neither the increase in food intake or weight gain, nor the increases in NPY and AgRP expression in the ARC typical of socially defeated WT mice. Exposure to CSDS also increases the incentive value of palatable food, an effect dependent on ghrelin actions on GHSR in catecholamine producing cells including dopamine cells in the VTA (41).

Similar effects have not been confirmed in female rodents. This might be because social defeat paradigms are more difficult to implement in females, but other social stress paradigms do point to social stressors as promoting weight gain and adiposity in females. For instance, social crowding increases adiposity in female mice despite causing decreases in food intake (126). There are a number of new models of female social defeat but these have not directly evaluated metabolic alterations, and studies are needed to address that gap. The exception is a recent paper, using a modified version of the chronic social defeat paradigm in which female experimental mice were laced with urine from males and introduced into the cage of an aggressive CD-1 mouse resident every day for 21 days. This protocol resulted in increases in body weight in stressed females compared to controls (102). It is unclear, however, whether this increase in weight gain is associated with increased caloric intake and/or are with increased ghrelin concentrations.

Social isolation stress has been linked to decreases in caloric intake and weight gain and the effects of 6-hours of isolation have been shown to decrease food intake in intact female mice to a greater extent than in males (100). Two hours of isolation in intact females is sufficient to increase circulating ghrelin, but this is associated with a reduction in food intake, compared to that of non-stressed controls. The anorectic effect of isolation was not eliminated by ovariectomy although ovariectomized females continued to eat more than controls. Overall, this suggests that estradiol continues to inhibit feeding during stress, regardless of increased ghrelin. Further, given that intact non stressed female mice in this study showed a robust response to ghrelin, these data also suggest that some other factor(s) may be suppressing the orexigenic response to ghrelin at this time (100). In contrast to the effects of acute social isolation, two weeks of social isolation stress induce significant increases in caloric intake in both male and female young but not old mice. Body weight was not altered by isolation in the younger groups but a decrease was observed in the older cohort. In addition to these age effects, some sex differences were observed in the response to isolation stress in the younger cohort: 1 week of isolation and an overnight fast were sufficient to increase circulating ghrelin levels in young males and increases in AgRP and NPY mRNA expression but these effects were not seen in young females. Two month old females did, however, show an increase in locomotor activity that was absent in males. Together, these data suggest that isolation stress induces an array of behavioral and metabolic responses some of which differ between males and females.

Chronic restraint stress has also been linked to changes in caloric intake, diet preference and metabolism together with obesity and insulin resistance (122). Zareian et al. (103) found that male rats exposed to 2 hours of restraint stress for a period of 2 weeks show increased plasma Des-Acyl ghrelin concentrations and caloric intake and a decrease in weight gain whereas female rats, increased their caloric intake and showed no changes in either Des-Acyl ghrelin or in weight gain (103). In another study, 3 weeks of 20 mins daily restraint induced elevated ghrelin concentrations accompanied by increased caloric intake and weight gain in both male and female rats and were still seen if females were ovariectomized. These data suggest thatstress induced increases in ghrelin do not depend on the presence of ovarian hormones although as only des-acyl ghrelin was measured whether these might influence ghrelin levels remains to be examined (101).

In primates, social stressors have also been associated with increased weight gain, changes in food preference, and bingeing (127, 128). Female rhesus monkeys that are subordinates in a colony tend to gain more weight than those that are higher ranking and that receive fewer social threats. These subordinate females also showed behavioral coping responses akin to emotional eating that include higher intake of a high sugar/high fat diet (127, 128). It is not known, however whether ghrelin concentrations are elevated in these individuals and how this might contribute to these effects. In humans, women report that stress increases their food consumption (129) and the consumption of highly palatable foods (130). Interestingly, female subjects exposed to the Trier stress paradigm, an experimental model to study social stress in humans, show rapid increases in plasma levels of ghrelin and these are associated with scores in a questionnaire measuring emotional eating (131). Using the same task, Raspopow et al. also found that ghrelin and cortisol levels were elevated also in anticipation of the social stressor (132). Because these studies were conducted only in females, it is unclear if similar changes occur in males, but these results support the notion that social stressors may increase ghrelin concentrations which could lead to eating as a coping mechanism to deal with the stressor.

## Conclusion

The discovery of ghrelin in 1999 was followed by an explotion of research to determine the biological role of this peptide and its potential for the treatment of metabolic disorders as well as disorders associated with altered emotional, cognitive and motivational states like depression, anxiety and addiction. Despite this enthusiasm, studies conducted on females were few, leaving a significant gap in knowledge on ghrelin biology. The studies discussed above suggest that, as in males, ghrelin increases feeding, promotes adiposity, and facilitates the oxidation of carbohydrates over fat. These effects, however, interact with those of the hormone estrogen, which opposes the metabolic effects of ghrelin (see **Figure 3**). In addition, it also seems that estradiol directly modulates the synthesis, secretion, or degradation of ghrelin, and the expression of the GHSR, perhaps as a feed-forward mechanism that allows for increased feeding after ovulation. Although there is some data showing estrogen-induced changes in GHSR expression within a particular subset of cells in the ARC, there is obviously a need for a broader examination of sex differences in GHSR expression in this and other GHSR expressing brain regions that are sexually differentiated, and on whether GHSR expression changes in these subsets of cells across the estrus cycle. Indirectly, estradiol may offset the orexigenic effects of ghrelin simply through its ability to potentiate the satiety-inducing pathways in the hypothalamus and brain stem and this may occur in a manner that independent from direct effects upon ghrelin secreting or ghrelin sensitice cells. A recent paper seems to substantiate this by showing that male mice show a more robust feeding and growth hormone response to exogenous ghrelin treatment than females, but females show a more robust response to leptin than males (133).

Ghrelin and estrogen not only have opposing effects on food intake but also on substrate utilization. This is reflected in increased oxygen consumption, that is particularly evident during the dark phase of the light/dark cycle of ovariectomized females receiving estradiol treatment (73). In addition, Giles et al. reported that RER declines during proestrus and estrus, supporting a role for estrogen in fuel utilization (74) and consistent with the hypothesis that estradiol promotes the utilization of fats as a source of energy. Conversely, ghrelin promotes the utilization of carbohydrates over fat (14). These differences could explain why *Ghsr^-/-^* females are more sensitive to the metabolic effects of GHSR deletion, than are *Ghsr^-/-^* mice males. It is possible that similar effects would be seen if comparisons were made between *Ghsr^-/-^* male mice and female mice in response to social stress. Clearly, more studies are required to determine the role of estrogen in regulating substrate metabolism in females, and how this interacts with the ghrelin system under different conditions that include acute and chronic stress.

Perhaps one of the most neglected areas of research is the interaction between estradiol and ghrelin in the modulation of reward circuitry and food seeking. In studies where effort based motivation has been examined, ghrelin increases while estradiol decreases food seeking. Paradoxically, the responses of dopamine cells in the VTA to the direct effects of these hormones are very similar and these may be the underlying substrate for how these hormones influence preference for palatable reinforcers and enhance the effects of psychostimulants like cocaine (49). Another potential site of ghrelin/estrogen interaction in the control of food seeking is the LHA. Antagonism of GHSR in this region produces greater effects on working for palatable food rewards in female rodents than in males and there is evidence that estrogen potentiates rewarding brain stimulation in this area (98). The specific neural phenotypes through which these effects are produced are currently unknown, as is whether GHSR and ER colocalize in this region. Given the heterogeneous nature of this hypothalamic region, it’s possible that ghrelin and/or estrogen affect other peptidergic neurons like melanocortin hormone, orexin or neurotensin all of which have been associated with the regulation of feeding and food reward (134, 135). Unfortunately little is known about sex differences in the expression of GHSR in the VTA, LH, NAc or other regions associated with food motivation, and/or whether cyclic hormonal fluctuations modulate the expression of GHSR in these regions. Furthermore, a more detailed examination of sex differences in different components of motivated behavioral in response to ghrelin and estrogen alone and in combination is required.

Finally, while emphasis has been placed on the interaction between estrogen and ghrelin, estrogen is not the only ovarian hormone that modulates food intake. Progesterone and its metabolite allopregnenalone stimulates food intake in male rodents (136). A recent study showed that allopregnanolone increased food intake when co-administered with ghrelin in male rats and significantly increased the orexigenic capabilities of ghrelin in a subset of rats in the study (137) possibly *via* its action as a positive modulator of the GABA_A_ receptor (138). The interaction between ghrelin and progesterone in females remains to be determined.

Overall, the research reviewed here supports clear sex differences in the regulation of homeostatic and hedonic feeding by ghrelin. To improve our understanding of the physiological effects of ghrelin, more research that includes females in experimental design is needed to close the gap in knowledge that exists in the study of ghrelin biology in females. These studies need to include close monitoring of the estrus cycle if we are to understand the complex relationship.

## Author Contributions

AS generated the first draft of the document. AA and BW complemented and edited the manuscript by adding their expertise in the subject of feeding and reproductive neuroendocrinology. All authors contributed to the article and approved the submitted version.

## Funding

This work was supported by funding from the Canadian Institutes for Health Research (CIHR) awarded to AA (Grant No. 178087).

## Conflict of Interest

The authors declare that the research was conducted in the absence of any commercial or financial relationships that could be construed as a potential conflict of interest.

## Publisher’s Note

All claims expressed in this article are solely those of the authors and do not necessarily represent those of their affiliated organizations, or those of the publisher, the editors and the reviewers. Any product that may be evaluated in this article, or claim that may be made by its manufacturer, is not guaranteed or endorsed by the publisher.

## Abbreviations

AgRP, Agouti-related protein; AMPK, Adenosine monophosphate protein kinase; ARC, Arcuate nucleus; BAT, Brown adipose thermogenesis; CART, Cocaine and amphetamine-regulated transcript; CCK, Cholecystokinin; CSDS, Chronic social defeat stress; DMH, Dorsomedial hypothalamus; ER, Estrogen receptor; Fos-ir, Fos immunoreactivity; GABA, γ-aminobutyric acid; GHSR, Growth hormone secretagogue receptor; GLP-1, Glucagon-like peptide 1; GOAT, Ghrelin o-acyltransferase; ICV, Intracerebroventricular; LH, Luteiniszing hormone; LHA, Lateral hypothalamus; α-MSH, α-melanocyte-stimulating hormone; m-TOR, Mammalian target of rapamycin; MCR3/4Melanocortin receptors 3 and 4; MPOA, Medial preoptic area; NPY, Neuropeptide Y; NTS, Nucleus of the solitary tract; POMC, Pro-opiomelanocortin; PVN, Paraventricular hypothalamus; UCP1, Uncoupling protein 1; VMH, Ventromedial hypothalamus; VTA, Ventral tegmental area; WT, Wild-type.
